# Sliding Graft Copolymer-Based Rubber Enables Enhanced Damping Performance and Mechanical Strength

**DOI:** 10.3390/polym18080900

**Published:** 2026-04-08

**Authors:** Kaijuan Li, Zhongxing Zhang, Wei Cheng, Guoxing Lin, Chengfei Liu

**Affiliations:** 1 Luoyang Ship Material Research Institute, Luoyang 471023, China; 2 Shaanxi Key Laboratory of Macromolecular Science and Technology, Xi’an Key Laboratory of Hybrid Luminescent Materials and Photonic Device, MOE Key Laboratory of Material Physics and Chemistry Under Extraordinary Conditions, School of Chemistry and Chemical Engineering, Northwestern Polytechnical University, Xi’an 710072, China

**Keywords:** sliding graft copolymer, rubber, damping performance

## Abstract

Noise pollution poses significant challenges to human health and quality of life; thus, high-performance damping materials are attracting increasing attention. Rubber has been extensively applied in these materials due to its viscoelasticity. However, the damping performance of these materials is often constrained by the intrinsically limited energy-dissipation capability of the polymer backbone, which lacks sound-absorbing functionalities. Herein, a cross-linked sliding graft copolymer (SGC) was incorporated into isobutylene-isoprene rubber (IIR) and chlorinated butyl rubber (ClIR) to fabricate high-strength damping elastomers. Unlike conventional covalently cross-linked polymers, the cross-linked SGC features mobile junctions, which can slide along the polyrotaxane backbone to redistribute and equalize chain tension, giving rise to the “pulley effect”. Benefiting from the intrinsically high energy-dissipation capability of SGC and the cooperative contribution of interfacial hydrogen bonding, the obtained SGC/IIR and SGC/ClIR blends exhibit both enhanced damping performance and mechanical properties. The synergistic improvement in damping capacity and mechanical robustness renders the SGC/rubber blends as promising candidates for advanced sound-absorption applications.

## 1. Introduction

Noise pollution arising from traffic, industrial machinery, and construction activities poses a serious concern for human health [1,2,3,4]. Acoustic vibration-attenuation systems based on energy-conversion principles have been developed to mitigate noise by dissipating acoustic energy or converting it into other forms, thereby reducing acoustic intensity and minimizing associated health risks [5,6,7,8,9]. The development of acoustic-absorption materials with high damping capabilities has attracted considerable attention in recent decades. For example, some porous materials such as cellular foams, nonwoven fabrics, and granular spheres have been extensively investigated [10,11,12]. These materials typically exhibit high sound-absorption coefficients and function by dissipating acoustic energy as the sound waves traverse their pore networks. This occurs via viscous friction along the pore walls and via thermal conduction at solid–air interfaces [13]. However, practically, such porous architectures are often plagued by structural fragility, arising from the limited mechanical flexibility and instability of the pore framework, which compromises damping performance and ultimately diminishes sound-absorption efficiency. To improve mechanical performance and structural stability, a range of polymer-based damping materials—including polymer foams, fiberglass, and natural polymers (e.g., cotton and flax)—have been developed [14,15,16]. In these systems, damping primarily functions through viscoelastic energy dissipation within the polymer matrix, thereby enabling more effective acoustic attenuation. Unfortunately, practical deployment is often constrained by their inherent drawbacks, including their pronounced hygroscopicity, poor corrosion resistance, and limited flame retardancy [13]. More critically, these systems’ insufficient mechanical strength and toughness severely impede their applicability in sound-absorbing applications. Collectively, these inherent shortcomings severely hinder their broader implementation in practical acoustic attenuation systems. Therefore, the development of damping materials that combine efficient energy dissipation with robust mechanical strength is essential for advanced noise-mitigation applications.

Owing to its high elasticity, superior mechanical strength, and low compression set, rubber, one of the most widely utilized elastomers, has been extensively employed across diverse engineering applications [17,18,19]. Elastomeric materials with good damping performance have been widely reported by leveraging rubber’s intrinsic viscoelastic energy dissipation within its polymeric matrices, providing a viable route toward enhanced sound-absorption efficiency [20]. However, because the polymer backbone lacks intrinsic sound-absorbing functionalities, energy attenuation in conventional rubber is largely governed by the viscoelastic dissipation pathway, which limits overall damping efficiency [20]. Therefore, introducing functional groups that can effectively couple with incident acoustic waves and dissipate their energy is an attractive strategy for increasing the intrinsic damping of rubber-like materials in noise-reduction applications. Recently, supramolecular interactions have been extensively exploited to engineer damping materials owing to their dynamic reversibility and inherent responsiveness to external stimuli [21,22,23,24,25,26,27]. Among the materials with this feature, sliding graft copolymers (SGCs) have attracted increasing attention owing to their polyrotaxane-based supramolecular architecture and distinctive mechanical behavior, endowing them with promising sound-absorption performance in high-damping applications [28,29,30,31]. In particular, cross-linked SGC materials exhibit low stress relaxation and a high damping factor (tan δ) over a broad frequency range [20]. The cross-linking junctions in SGCs can slide freely along the polyrotaxane backbone to redistribute and equalize the tension of the threaded polymer chains, a phenomenon referred to as the “pulley effect”, enabling efficient energy dissipation under dynamic loading [20,32,33,34]. Therefore, incorporating SGCs as additives into rubber is expected to enhance both damping capacity and mechanical flexibility, thereby enabling more effective noise attenuation.

**Scheme 1:**
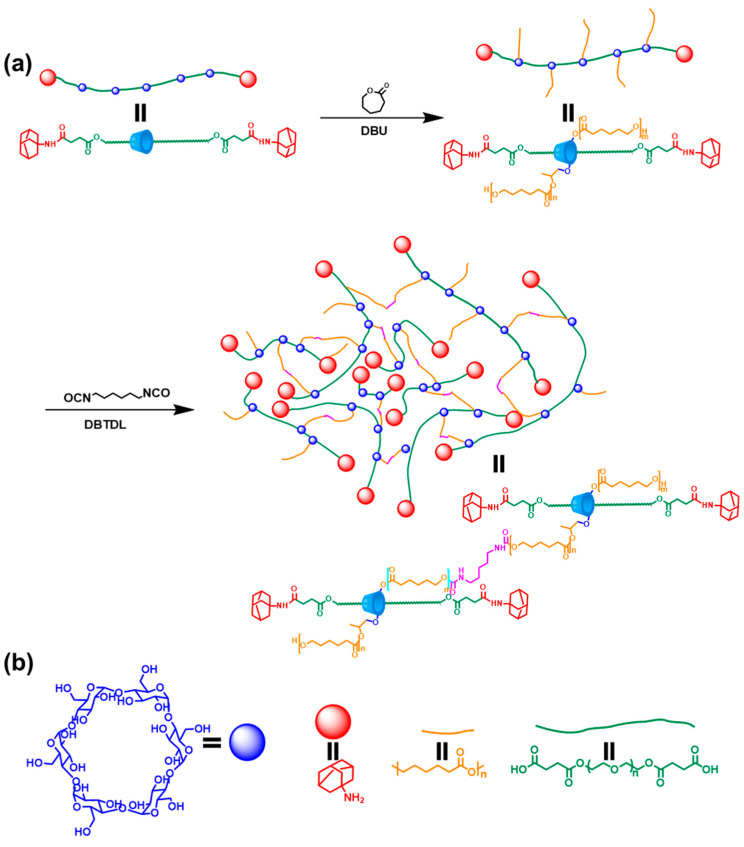
(**a**) Schematic illustration of the cross-linking process of SGC. (**b**) Chemical structures of α-cyclodextrins, poly-ε-caprolactone and poly(ethylene glycol).

Based on these considerations, we prepared an SGC and blended it with two representative high-performance elastomers—isobutylene–isoprene rubber (IR) and chlorinated isobutylene–isoprene rubber (ClIR)—to obtain SGC/IIR and SGC/ClIR blends that simultaneously exhibit a high damping capacity and enhanced mechanical strength. In this work, we systematically investigated the microstructure, damping behavior, and mechanical performance of these SGC/rubber blends and established correlations between their unique architectures and macroscopic properties. The SGC network was first prepared by cross-linking terminal groups of PCL chains with hexamethylene diisocyanate (HMDI) (Scheme 1). Notably, the cross-linked SGC differs from conventional covalently cross-linked polymers in both its physical and chemical behavior, as its junctions can slide freely along the polyrotaxane backbone to redistribute and equalize chain tension via the “pulley effect”. Dynamic thermomechanical analysis (DMA) revealed that both SGC/IR and SGC/ClIR blends exhibit enhanced damping properties over the investigated frequency range relative to neat IR and ClIR, respectively. In addition, the hydroxyl groups of the SGC can form hydrogen-bond interactions with the chlorine-containing moieties on the ClIR chains, strengthening interfacial affinity and enabling more efficient stress transfer. Compared with previously reported systems, the SGC/CIIR blends exhibit a markedly enhanced tensile strength of up to 32.9 MPa, and their damping factor increases significantly with increasing vibration frequencies. This enhanced interfacial interaction rationalizes the superior mechanical performance of SGC/ClIR compared with SGC/IR. In this design, covalent cross-linking is integrated with dynamic supramolecular interactions, preserving the mechanical robustness of conventional polymers while introducing stimuli-responsive adaptability and thereby conferring superior energy-dissipation capability.

## 2. Materials and Methods

### 2.1. Materials

α-Cyclodextrin (α-CD, content > 99%) was purchased from Adamas-beta. Poly(ethylene glycol) (PEG, *M*_n_ = 20,000) was purchased from Kelong Chemical Co. (Chengdu, China). ε-Caprolactone (ε-CL) was purchased from Aldrich; Succinic anhydride was purchased from China National Pharmaceutical Group Co., Ltd. (Haidian District, Beijing, China). Hexamethylene diisocyanate (HMDI), 1,8-Diazabicyclo [5.4.0]undec-7-ene (DBU), and dibutyltin dilaurate (DBTDL) were purchased from Tokyo Chemical Industry Co., Ltd. (Tokyo, Japan). Other chemicals were purchased from energy chemical. All reagents were of experimental grade and used without further purification.

### 2.2. Structure Characterization Methods

^1^H NMR spectra were obtained on a Bruker Avance 400 spectrometer, (Karlsruhe, Germany). X-ray Photoelectron Spectroscopy (XPS) measurements were obtained on a Kratos AXIS Ultra DLD, (Manchester, UK). The tensile data was obtained using an electronic universal testing machine CMT6303. The dynamic mechanical properties of rubber materials were measured on a dynamic mechanical analyzer (DMA, VA3000, 01dB-Metravib, Paris, France). DMA was conducted with a vibration amplitude of 10 μm, and a heating rate of 5 °C min^−1^. Fourier transform infrared spectroscopy (FTIR) measurements were performed on a Bruker Model Tensor 27 FTIR spectrometer, (Karlsruhe, German). Differential scanning calorimeter (DSC) data were obtained on a DSC1 Mettler Toledo, (Zurich, Switzerland). DSC was performed at a heating rate of 10 °C min^−1^. Gel permeation chromatography (GPC) data were obtained on Waters 515 (Milford, MA, USA). The mixing of the sample was completed by a torque rheometer Haake Poly Lab OS. The opening and refining of rubber were completed by the opening and refining machine. The sample for transmission electron microscopy (TEM) measurement was prepared using a FEI Talos F200X instrument, (Hillsboro, OR, USA).

### 2.3. The Method of SGC/Rubber Blends

Raw rubber and SGC were charged into an internal mixer and blended until the temperature reached 60 °C. A mixture of magnesium oxide, zinc oxide, and stearic acid was then added, and mixing was continued for 2 min. Carbon black was subsequently introduced and mixed for an additional 4 min. Following this, accelerators TMTD, DM, and dibutyl phthalate were added, and the mixture was compounded for 2 min. The resulting rubber compound was then discharged from the mixer. The compound was transferred to a two-roll mill and allowed to band on the rolls. Sulfur was added, and the material was cut and folded to form 4–5 triangular bags until a smooth, bubble-free sheet was obtained. The sheet was then removed from the mill. Finally, the sample was subjected to compression molding in a flat vulcanizing press at a designated temperature and time to complete the vulcanization process. The vulcanized rubber composite was obtained after demolding.

## 3. Results and Discussion

### 3.1. Preparation of SGC/Rubber Blends

The SGC was synthesized by threading α-cyclodextrin rings onto a carboxylic acid-functionalized PEG backbone, followed by end-capping with adamantane amine and grafting of PCL side chains. Subsequent cross-linking with HMDI affords the corresponding slide-ring elastomer network. According to previous work, the polyrotaxane was prepared from a poly (ethylene glycol) (PEG) backbone threaded with multiple α-cyclodextrin (α-CD) rings and end-capped with bulky adamantane groups [32,33,34]. Synthetic procedures and characterization results are shown in detail in Figures S1–S3. The molecular weight of polyrotaxane, measured by GPC and ^1^H NMR, is shown in Figures S1 and S2, respectively, further confirming its successful synthesis. The α-CD rings in the polyrotaxane were subsequently grafted with linear poly(ε-caprolactone) (PCL) side chains, and the SGC network was established by cross-linking the PCL chain termini with hexamethylene diisocyanate (HMDI). Figure S4 shows the ^1^H NMR spectrum of the cross-linked SGC. Pronounced resonances from the PCL side-chain protons are observed at 1.39, 1.64, 2.26, and 3.98 ppm, while signals attributable to the polyrotaxane backbone are also detected, which indicates the successful synthesis of cross-linked SGC. The cross-linked SGC was melt-compounded with the rubber matrix using an internal mixer to ensure a uniform dispersion and intimate interfacial contact. Finally, the resulting mixtures were then subjected to sulfur vulcanization to establish the final cross-linked network present in the SGC/rubber blends.

**Figure 1:**
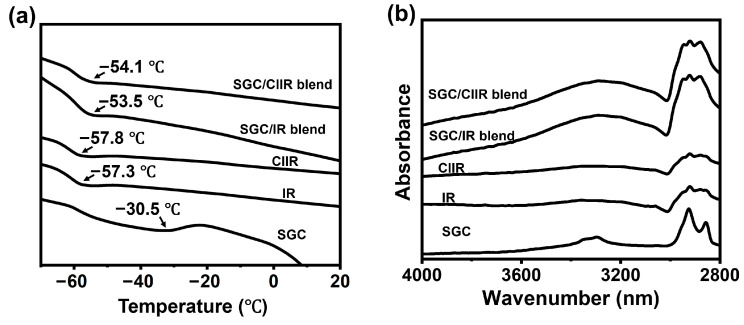
(**a**) DSC curves of SGC, IR, ClIR, SGC/IR and SGC/ClIR blends. (**b**) FTIR spectra of SGC, IR, CIIR, SGC/IR and SGC/ClIR blends, in the OH group stretching vibration region.

To evaluate the miscibility and phase interactions in the SGC/rubber blends, differential scanning calorimetry (DSC) was employed. Figure 1a shows the DSC curves of SGC, IIR, ClIR, and the SGC/IIR and SGC/ClIR blends. Notably, both the SGC/IIR and SGC/ClIR blends exhibit a single glass transition temperature (T_g_), corroborating the favorable miscibility of SGC with both the IR and CIIR phases. The DSC results also show that the T_g_ values for neat SGC, IR, and ClIR are −30.5 °C, −57.3 °C, and −57.8 °C, respectively. SGC chain segments exhibit higher mobility than those of IIR and ClIR; thus, adding more SGC would be expected to reduce the T_g_ of the SGC/IIR and SGC/ClIR blends. However, the measured T_g_ values for the SGC/IIR and SGC/ClIR blends are −53.5 °C, and −54.1 °C, respectively, which are unexpectedly higher than those of the corresponding single-component phases. This anomalous increase can be attributed to the formation of hydrogen-bond interactions between the SGC and rubber chains, restricting segmental mobility and thereby elevating the T_g_. FTIR measurements were performed to further verify the presence of hydrogen-bond interactions between the SGC and rubber chains. Figure 1b shows the FTIR spectra of SGC, IIR, ClIR, and the SGC/IIR and SGC/ClIR blends. In the SGC spectrum, the broad absorption band at approximately at ∼3379 cm^−1^ is assigned to the stretching vibrations of hydroxyl groups, arising from both free OH groups and hydrogen-bonded OH species. The hydrogen-bonded species are associated with intermolecular OH···OH interactions as well as OH···C=O hydrogen bonding. In the spectrum of the SGC/ClIR blend, the absorption band corresponding to hydrogen-bonded groups shifts from 3379 to 3366 cm^−1^, indicating strengthened intermolecular interactions. This redshift is ascribed to hydrogen-bond formation between the hydroxyl groups of SGC and the chlorine-containing moieties of ClIR. These results further corroborate the observations of strengthened interfacial interactions within the blend systems.

**Figure 2:**
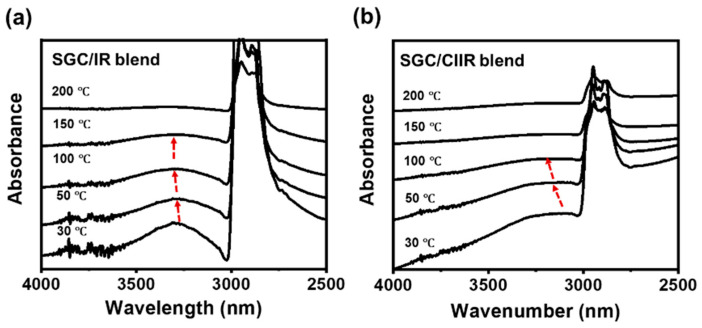
In situ FTIR spectra of SGC/IR (**a**) and SGC/ClIR blends (**b**) under different temperatures (from 30 °C to 200 °C).

It is well established that hydrogen-bonding interactions are thermodynamically sensitive to temperature. As the temperature increases, the hydrogen/bond interactions are progressively weakened. Accordingly, the temperature-dependent diffuse reflectance FTIR measurements were conducted to further study the existence and thermal responsiveness of hydrogen-bonding interactions in the SGC/rubber blends. Figure 2a demonstrates the temperature-dependent evolution of the OH stretching region for the SGC/IR blend. As the temperature increases, the bands associated with hydrogen-bonded OH groups progressively weaken and shift to higher wavenumbers, consistent with thermally induced hydrogen-bond dissociation. Similar temperature-dependent behavior is also observed in the SGC/ClIR blend, further confirming the presence of hydrogen-bonding interactions in these systems (Figure 2b). It is noteworthy that, because IR lacks chlorine-containing functionalities, the corresponding temperature-induced changes are less pronounced than those of the SGC/CIIR blends, indicating its weaker interfacial hydrogen bonding.

### 3.2. Microstructure of SGC/Rubber Blends

**Figure 3:**
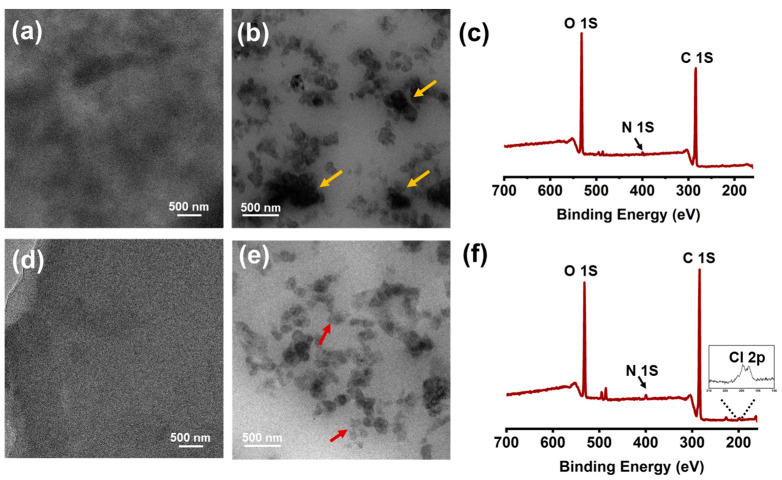
TEM images of (**a**) IR, (**b**) SGC/IR blend, (**c**) ClIR, (**d**) SGC/ClIR blend. XPS of (**e**) SGC/IR blend and (**f**) SGC/CIIR blend.

After confirming the successful formation of the SGC/rubber blends, the internal macroscopic structure was systematically investigated. Transmission electron microscopy (TEM) was first employed to directly visualize the internal microstructure of the SGC/rubber blends, with particular emphasis on phase distribution and structural homogeneity. Prior to TEM analysis, the SGC/rubber blends samples were cryo-ultramicrotomed using a glass knife on the same ultramicrotome to ensure consistent sectioning conditions. Subsequently, the resulting ultrathin sections were stained with OsO_4_ for 10 min to selectively enhance the contrast of the SGC phase prior to observation; consequently, the bright domains are assigned to the dispersed SGC phase, whereas the dark continuous matrix corresponds to the rubber. This distinct contrast clearly delineates a phase-separated morphology, enabling direct visualization of the dispersion state and interfacial characteristics within these blend systems. In Figure 3b, the nanometer-sized SGC domains are uniformly distributed throughout the ClIR matrix with a diameter (*D*_av_, TEM) of 221.6 ± 3.8 nm, indicative of good dispersion and interfacial compatibility. The formation of such a distinctive microstructure can be ascribed to the favorable compatibility between SGC and ClIR. This interpretation is supported by their strong mutual affinity, a portion of the PCL chains in SGC can diffuse into the ClIR phase and form entanglements with ClIR macromolecules, thereby strengthening interfacial adhesion. However, owing to the intrinsically low mechanical strength of SGC, portions of the dispersed phase are readily thinned or even detached during ultramicrotoming, leading to localized inhomogeneities in the apparent distribution of SGC domains in certain regions, which is theoretically reasonable (Figure 3e). In contrast, irregular aggregates and other heterogeneous morphologies are observed in the SGC/IR blend, indicating poor interfacial compatibility between SGC and IR. This behavior likely arises from the low polarity of IR, which limits effective interdiffusion and interactions with the PCL chains in SGC.

Moreover, to further elucidate the compositional characteristics and interfacial structure of SGC/rubber blends, the surface elemental composition and corresponding oxidation states of the SGC/IR and SGC/ClIR blends were analyzed by X-ray photoelectron spectroscopy (XPS). As shown in Figure 3c, the survey spectrum of the SGC/IR blend displays all expected constituent elements, including carbon (C 1s), nitrogen (N 1s), and oxygen (O 1s), thereby confirming the successful incorporation and coexistence of the respective components within the blend. Meanwhile, the survey spectrum of the SGC/ClIR blend (Figure 3e) exhibits a characteristic Cl 2p signal with a binding energy centered at 200.1 eV, unequivocally confirming the incorporation of chlorine-containing ClIR segments. Taken together, XPS results corroborate the successful formation of the SGC/Rubber blends and verify the coexistence of both components within the hybrid structure.

### 3.3. Damping Performance of SGC/Rubber Blends

**Figure 4:**
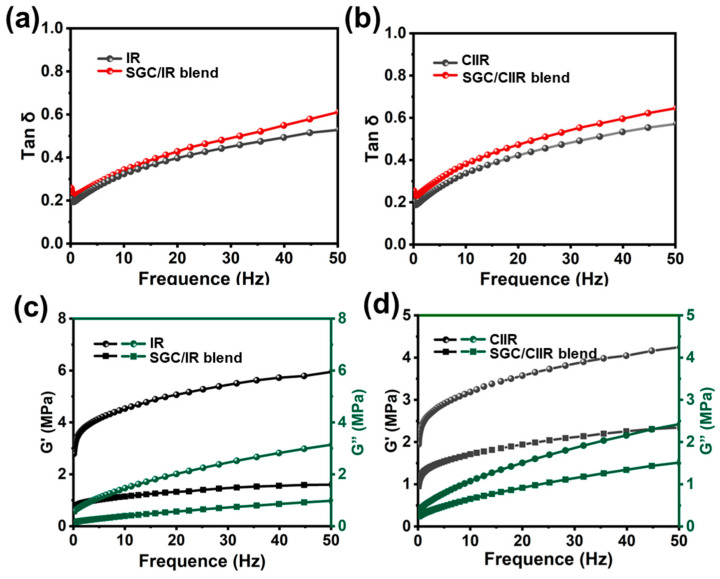
Variation in tan δ with dynamic frequency sweep for SGC/IR blend (**a**) and SGC/ClIR blend (**b**); Frequency-sweep rheological curves of SGC/IR blend (**c**) and SGC/ClIR blend (**d**) from 0 to 50 Hz.

To evaluate the damping performance of SGC/rubber blends, the loss factor (tan δ = G″/G′) was first analyzed as a function of frequency via rheological measurements. Dynamic thermomechanical analysis (DMA) tests were conducted to investigate tan δ variation in neat IR and ClIR, as well as the SGC/IR and SGC/ClIR blends, under frequency conditions. As shown in Figure 4a, both the SGC/IR and SGC/ClIR blends exhibit enhanced damping properties over the investigated frequency range compared with neat IR and ClIR. The elevated tan δ values can be attributed to the “pulley effect” enabled by dynamic interactions between the SGC and IR/ClIR rubber. Under mechanical vibration, the cross-linked junctions within the SGC network can slide along the polymer backbone, increasing internal friction among the chains and enhancing energy dissipation, which leads to higher tan δ values. The tan δ values of the SGC/ClIR blend are higher than those of the SGC/IR blend. This interesting phenomenon may be attributed to the stronger hydrogen-bond interactions between the SGC and ClIR chains, which promote interfacial energy dissipation and thus improve the damping performance. Collectively, these results demonstrate that the SGC/ClIR blends exhibit superior damping capability and may hold considerable promise for sound-absorption applications.

Impact-stiffening behavior, characterized by strain-rate-induced phase transitions from compliant to rigid states, is highly desirable for protective damping materials in sound-absorption and noise-mitigation applications. The transient physical interactions among supramolecular polymer chains in the SGC/rubber blends are strongly strain-rate-sensitive, enabling a rapid soft-to-hard transition under impact loading. As shown in Figure S6, although slight hand pressure deformed the SGC/rubber blends, they remained virtually undeformed when subjected to a heavy blow from a hammer or substantial weight. The impact-stiffening behavior of these blends was quantitatively assessed through rheological tests across a frequency range of 1–50 Hz (Figure 4c,d), revealing prominent impact-stiffening behavior in both systems. The most pronounced response was observed for the SGC/ClIR blends; the storage modulus (G′) increased dramatically from 0.78 MPa at 0.01 Hz to 2.49 MPa at 50 Hz, representing an impressive enhancement of approximately 3.1-fold (Figure S7). Collectively, these results demonstrate that the SGC/ClIR blends exhibit excellent damping performance coupled with pronounced impact-stiffening behavior.

### 3.4. Mechanical Property of SGC/Rubber Blends

**Figure 5:**
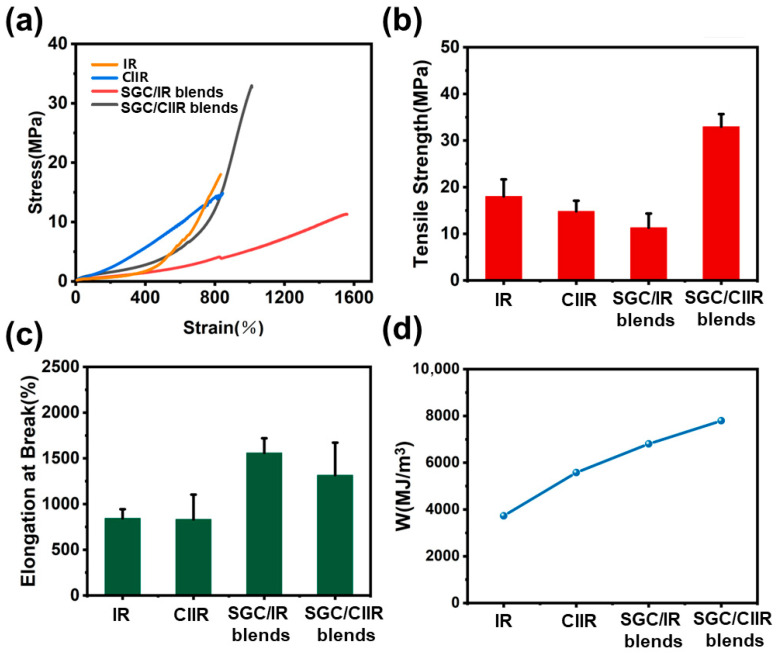
Stress–strain curves (**a**) and tensile strength (**b**) of IR, ClIR, SGC/IR blend and SGC/ClIR blend; (**c**) Elongation at break of SGC/IR and SGC/ClIR blends; (**d**) Fracture energy of SGC/IR and SGC/ClIR blends. Data are represented as average ± standard error (n = 3).

Monotonic tensile stress–strain measurements were conducted to evaluate the mechanical properties of the SGC/IR and SGC/ClIR blends. As shown in Figure 5a, neat ClIR exhibits a tensile strength of approximately 14.8 MPa, whereas the weakly cross-linked SGC displays poor mechanical performance, with a tensile strength of only ~0.05 MPa. Notably, the SGC/ClIR blends demonstrate an enhanced tensile strength of ~32.9 MPa, surpassing that of neat ClIR and markedly exceeding that of SGC alone (Figure 5b). This performance improvement indicates that the SGC/ClIR blend possesses superior mechanical properties, which can be attributed to the favorable compatibility between SGC and ClIR. During tensile deformation, the cross-linked SGC network in the SGC/ClIR blend acts as a dynamic reinforcing phase, modulating stress transfer and enhancing the load-bearing capability. In contrast, the SGC/IR blend exhibits a lower tensile strength than neat IR. This deterioration in mechanical performance likely arises from phase separation due to poor interfacial compatibility between SGC and IR, which weakens stress transfer efficiency and ultimately reduces the tensile strength.

Interestingly, the elongation at break values for both the SGC/IR and SGC/ClIR blends are significantly enhanced compared with those of neat IR and ClIR, respectively (Figure 5c). This improvement suggests that incorporating SGC enhances ductility and toughness by introducing a “pulley effect” at the microstructural level and strengthening interfacial adhesion with the rubber matrix, thereby increasing ductility and toughness. As a result, the blends exhibit improved flexibility and can withstand greater deformation without premature fracture. The fracture energies of the SGC/IR and SGC/ClIR blends were determined by integrating the area under the corresponding stress–strain curves. As shown in Figure 5d, the fracture energy of the SGC/ClIR blend increased markedly relative to that of neat ClIR, indicating that the incorporation of the SGC significantly enhanced energy dissipation during mechanical deformation. The elevated fracture energy of the SGC/ClIR blend implies that it can withstand substantial plastic deformation under applied stress by dissipating mechanical energy and mitigating stress accumulation.

## 4. Conclusions

In conclusion, we have successfully fabricated SGC-based rubber blends that exhibit both enhanced damping and mechanical characteristics. For the SGC/ClIR blend, the formation of hydrogen-bond interactions between the hydroxyl groups of SGC and the chlorine-containing moieties of ClIR was experimentally verified. The tan δ value of the SGC/ClIR blend was markedly higher than that of neat ClIR, indicating substantially improved damping performance. Both the tensile strength and fracture energy were also substantially enhanced relative to pristine ClIR. The experimental results reveal a pronounced strain-induced chain orientation in the blends, which can be attributed to the SGC-enabled “pulley effect” coupled with the dynamic rupture and reformation of interfacial hydrogen bonds. The unique combination of high-damping capability and enhanced mechanical strength highlights the considerable potential of the SGC/ClIR blend for application in high-performance sound-absorption applications. The obtained high-damping elastomers could play an important role in industrial vibration and noise mitigation, with clear relevance in seismic isolation, aerospace systems, and large-scale civil infrastructure such as bridges.

## Data Availability

The original contributions presented in this study are included in the article/Supplementary Materials. Further inquiries can be directed to the corresponding authors.

## Supplementary Materials

The following supporting information can be downloaded at: https://www.mdpi.com/article/10.3390/polym18080900/s1, Scheme S1. Synthetic route to polyrotaxane composed of α-cyclodextrin and poly(ethylene glycol). Figure S1. ^1^H NMR spectrum (400 MHz, DMSO, RT) recorded for polyrotaxane. Figure S2. GPC traces of polyrotaxane (Water). Figure S3. ^1^H NMR spectrum (400 MHz, DMSO, RT) recorded for PCL-g-PR. Figure S4. ^1^H NMR spectrum (400 MHz, DMSO, RT) recorded for SGC. Figure S5. GPC traces of SGC (water). Figure S6. Demonstration of the impact-stiffening behavior under gentle pressing or heavily impacting the SGC/ClIR blend. Figure S7. Impact-stiffening response reflected by the ratio of storage modulus at 50 Hz to that at 0.01 Hz.
