# Supplementary material for: Sliding Graft Copolymer-Based Rubber Enables Enhanced Damping Performance and Mechanical Strength
Source: Polymers (Basel). 2026 Apr 8;18(8):900. doi: 10.3390/polym18080900 (PMC13119547; doi:10.3390/polym18080900)
Supplement: Supplementary file 1 [file polymers-18-00900-s001.zip › polymers-4219100-supplementary.pdf]

Sliding Graft Copolymer-Based Rubber Enables Enhanced Damping Performance and Mechanical Strength

[1]Kaijuan Li[+], [2]Zhongxing Zhang[+], [1]Wei Cheng, [1]Guoxing Lin[*], [2]Chengfei Liu[*]

[1] Luoyang ship Material Research Institute, Luoyang 471000, China.

[2] Shaanxi Key Laboratory of Macromolecular Science and Technology,

Xi'an Key Laboratory of Hybrid Luminescent Materials and Photonic Device,

MOE Key Laboratory of Material Physics and Chemistry under Extraordinary Conditions, School of Chemistry and Chemical Engineering,

Northwestern Polytechnical University,

Xi'an, 710072, Shaanxi P. R. China

Email: linguox@163.com; liuchengfei@nwpu.edu.cn

[+]K. Li. and Z. Zhang contributed equally to this work.

Table of Contents

1. Synthesis of SGC

2. Preparation of Vulcanized Rubber Composite

3. Damping Performance of SGC/Rubber Blends

4. Reference

1. Synthesis of SGC

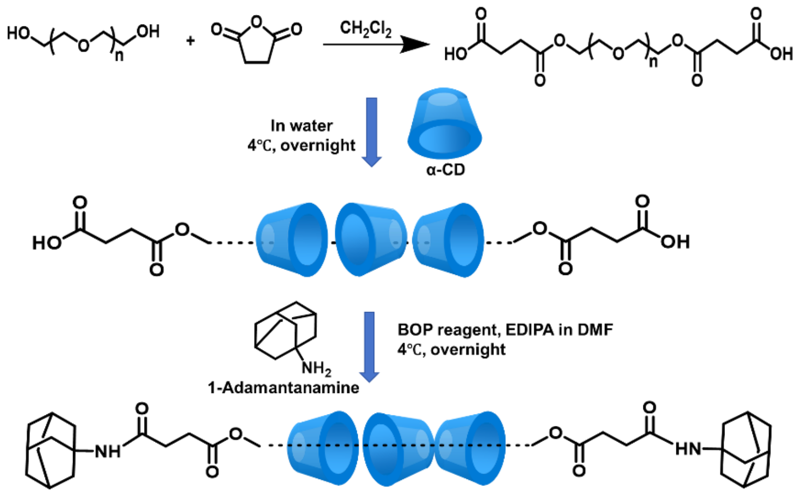

Scheme S1. Synthetic route to polyrotaxane composed of α-cyclodextrin and poly(ethylene glycol).

1.1 Synthesis of PEG-COOH

Polyethylene glycol (50.00 g) and succinic anhydride (6.25 g) were dispersed and dissolved in 100 mL of anhydrous dichloromethane. Triethylamine (3 mL) was then added dropwise to the mixture, and the resulting solution was heated at 80 °C under reflux in an oil bath for 36 h. After completion of the reaction, the mixture was washed repeatedly with excess acetone until a white precipitate formed. The solvent was removed to afford the product as a white solid.

1.2 Synthesis of polyrotaxane[1]

The obtained PEG-COOH (3.0 g, 8.6×10^{-5} mol) and α-cyclodextrin (α-CD, 12 g, 1.2×10^{-2} mol) were dissolved in water (100 mL), and the solution was stored in a refrigerator overnight to afford a white paste-like inclusion complex. The resulting freeze-dried complex (14 g) was mixed with adamantanamine (0.16 g, 1.1×10^{-3} mol), (benzotriazol-1-yloxy)tris(dimethylamino)phosphonium hexafluorophosphate (BOP reagent, 0.48 g, 1.1×10^{-3} mol) , and ethyldiisopropylamine (EDIPA, 0.19 mL, 1.2×10^{-3} mol) dissolved in anhydrous DMF (100 mL). The slurry-like mixture was allowed to react at 4 °C overnight. The mixture was subsequently washed repeatedly with ethanol and freeze-dried to afford the product as a white solid polyrotaxane.

1.3 Synthesis of poly(ε-caprolactone)-grafted polyrotaxane[2]

PR (9.5 g) was dispersed in ε-caprolactone (95 mL), followed by the addition of DBU (4.75 mL).

The mixture was stirred at 60 °C for 4 h. After cooling to room temperature, acetic acid (4.5 mL) was added to neutralize the solution. The resulting mixture was then poured into ethanol (1000 mL) to precipitate the product, which was collected by centrifugation and washed with ethanol. The obtained solid was dried under vacuum to afford the poly(ε-caprolactone)-grafted polyrotaxane (PCL-g-PR) as a white solid.

1.4 Synthesis of SGC[3]

A crosslinked SGC film was prepared by dissolving the obtained PCL-g-PR (250 mg) in anhydrous toluene (5 mL), followed by the addition of dibutyltin dilaurate (DBTDL, 50 μL) and hexamethylene diisocyanate (HMDI, 32 μL). The mixture was cast onto a Teflon petri dish (50 mm in diameter) and allowed to dry overnight at room temperature. Crosslinking with HMDI occurred during toluene evaporation, affording a semi-transparent thin film.

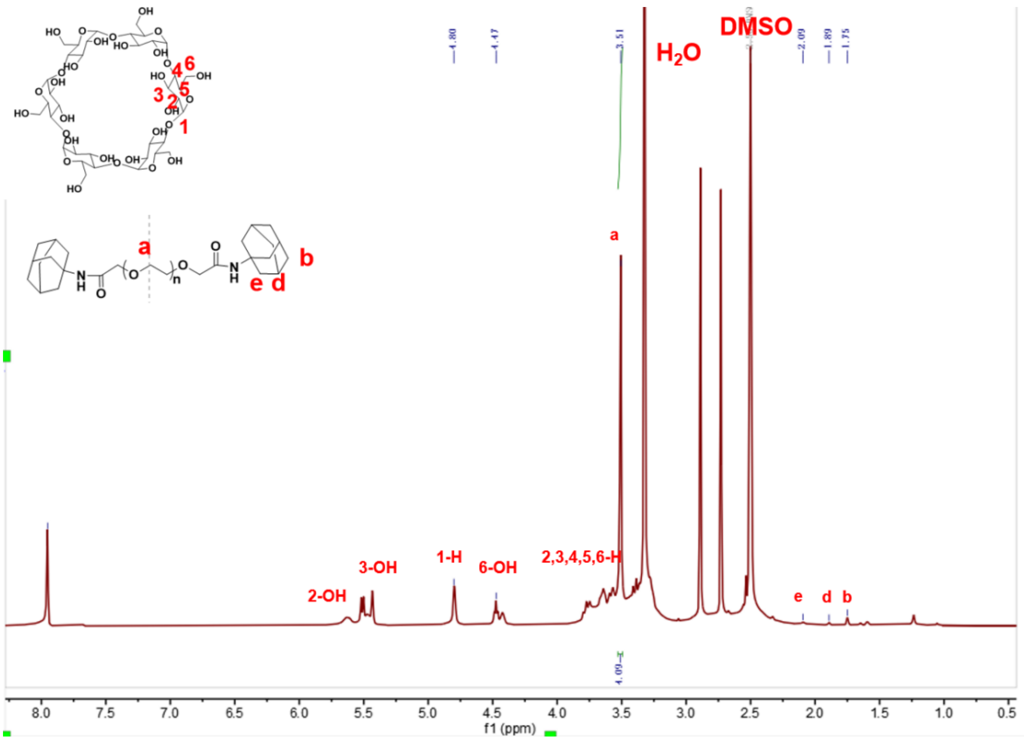

Figure S1. ^{1}H NMR spectrum (400 MHz, DMSO, RT) recorded for polyrotaxane .

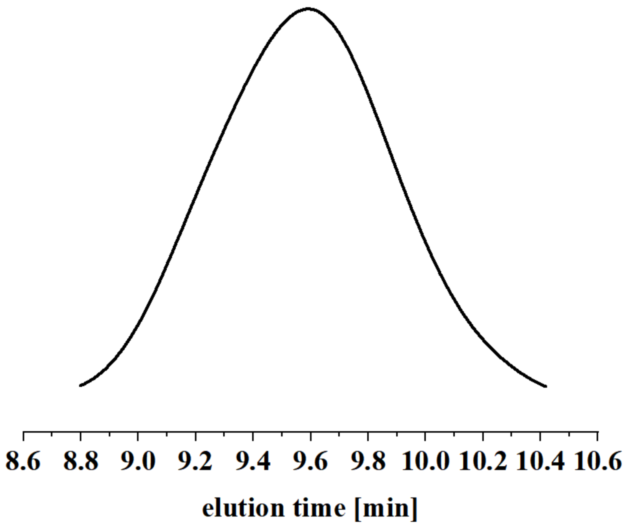

Figure S2. GPC traces of polyrotaxane (Water)

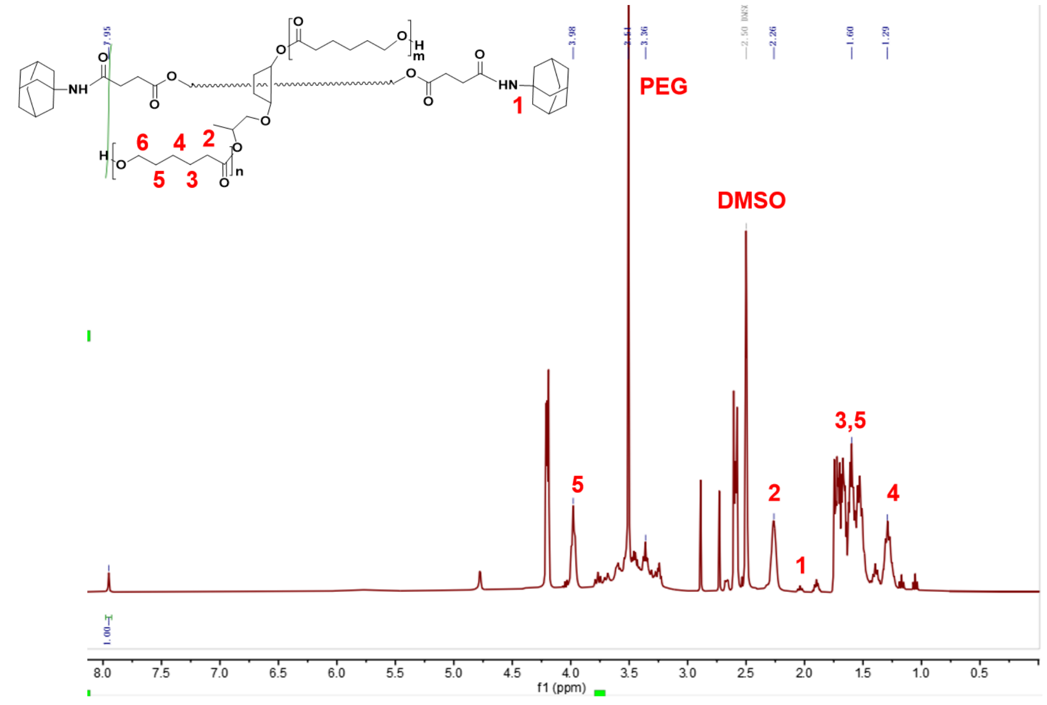

Figure S3. ^{1}H NMR spectrum (400 MHz, DMSO, RT) recorded for PCL-g-PR.

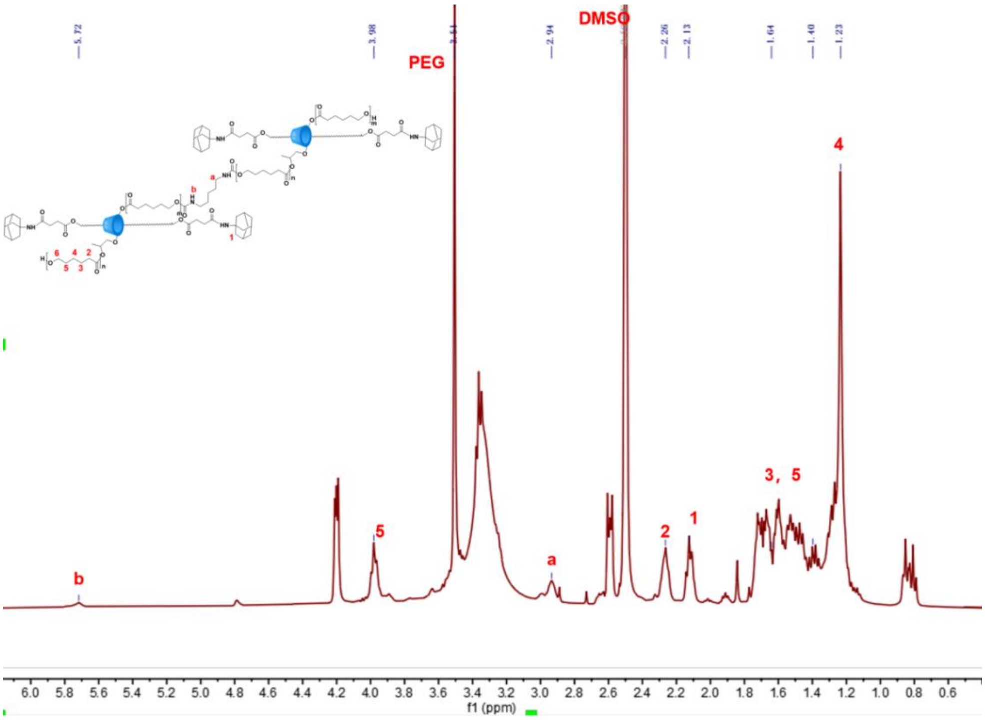

Figure S4. ^{1}H NMR spectrum (400 MHz, DMSO, RT) recorded for SGC.

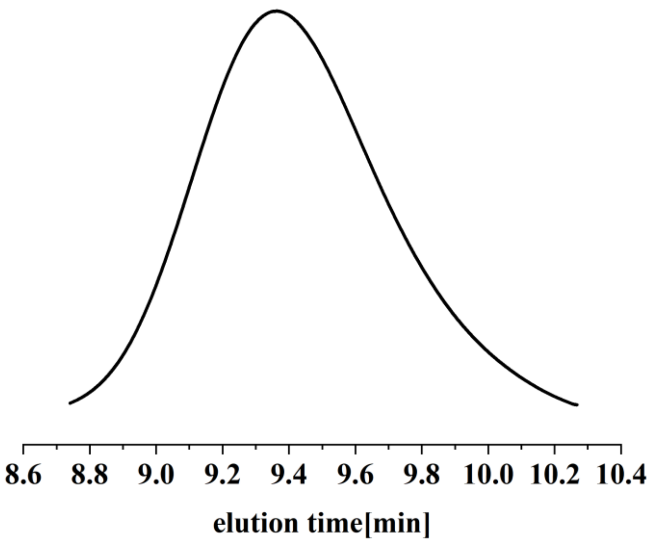

Figure S5. GPC traces of SGC (water)

2. Preparation of Vulcanized Rubber Composite

Raw rubber and SGC were charged into an internal mixer and blended until the temperature reached 60 °C. A mixture of magnesium oxide, zinc oxide, and stearic acid was then added, and mixing was continued for 2 min. Carbon black was subsequently introduced and mixed for an additional 4 min. Following this, accelerators TMTD, DM, and dibutyl phthalate were added, and the mixture was compounded for 2 min. The resulting rubber compound was then discharged from the mixer. The compound was transferred to a two-roll mill and allowed to band on the rolls. Sulfur was added, and the material was cut and folded to form 4-5 triangular bags until a smooth, bubble-free sheet was obtained. The sheet was then removed from the mill. Finally, the sample was subjected to compression molding in a flat vulcanizing press at a designated temperature and time to complete the vulcanization process. The vulcanized rubber composite was obtained after demolding.

3. Damping Performance of SGC/Rubber Blends

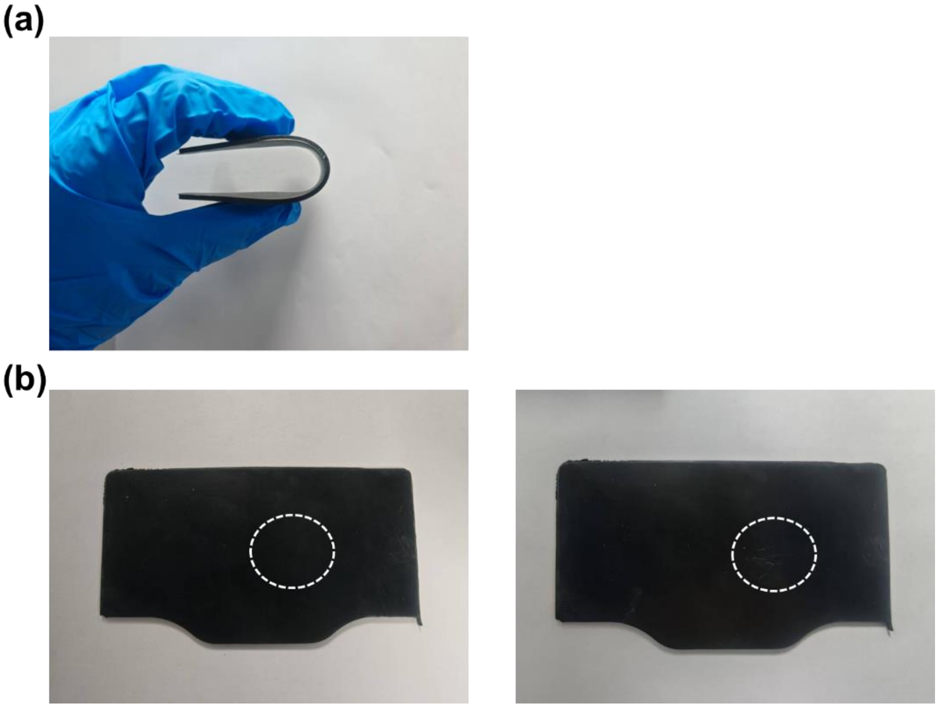

Figure S6. Demonstration of the impact-stiffening behavior under gentle pressing or heavily impacting the SGC/ClIR blend.

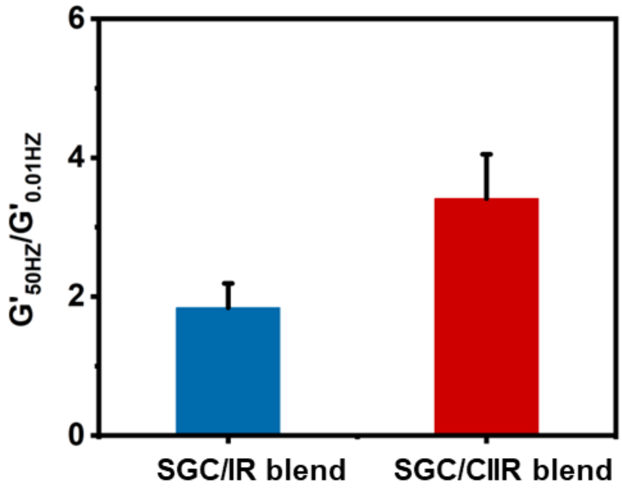

Figure S7. Impact-stiffening response reflected by the ratio of storage modulus at 50 Hz to that at 0.01 Hz.

4. References

[1] Araki, J.; Kataoka, T.; Ito, K. Preparation of a "sliding graft copolymer", an organic solvent-soluble polyrotaxane containing mobile side chains, and its application for a crosslinked elastomeric supramolecular film. *Soft Matter* **2008**, *4* (2), 245-249.

[2] Okumura, Y.; Ito, K. The Polyrotaxane Gel: A Topological Gel by Figure-of-Eight Cross-links. *Adv. Mater.* **2001**, *13*, 7.

[3] Zhao, X.Y.; Niu, K.J.; Xu, Y.; Peng, Z.; Jia, L.; Hui, D.; Zhang, L.Q. Morphology and performance of NR/NBR/ENR ternary rubber composites. *Composites, Part B* **2016**, *107*, 106−112.

[4] Wang, J.J.; Zhao, X.Y.; Wang, W.C.; Geng, X.Y.; Zhang, L.Q.; Guo, B.C.; Nishi, T.; Hu, G.H. Significantly Improving Strength and Damping Performance of Nitrile Rubber via Incorporating Sliding Graft Copolymer. *Ind. Eng. Chem. Res.* **2018**, 57, 16692-16700.
